# A New Sauropod Dinosaur from the Middle Jurassic of the United Kingdom

**DOI:** 10.1371/journal.pone.0128107

**Published:** 2015-06-01

**Authors:** Phillip L. Manning, Victoria M. Egerton, Mike Romano

**Affiliations:** 1 School of Earth, Atmospheric and Environmental Sciences, Interdisciplinary Centre for Ancient Life, University of Manchester, Oxford Road, Manchester, United Kingdom; 2 Independent Researcher, 14 Green Lane, Dronfield, Sheffield, United Kingdom; Perot Museum of Nature and Science, UNITED STATES

## Abstract

A new record of a sauropodomorph dinosaur is here described from the Middle Jurassic (Aalenian) Saltwick Formation of Whitby (Yorkshire), UK. A single caudal vertebra represents an early sauropodomorph and signifies the earliest recognised eusauropod dinosaur from the United Kingdom. The absence of pleurocoels and a narrow, dorsoventrally deep, but craniocaudally short centrum, suggests a primitive sauropodomorph. Distinct spinopostzygopophyseal laminae rise from the lateral margins of the postzygapophyses and pass caudally along what remains of the neural spine, a character unique to a subgroup of sauropods that includes *Barapasaurus*, *Omeisaurus* and other neosauropods and eusauropods. The lack of phylogenetically robust characters in sauropod caudal vertebrae usually makes it difficult to establish affinities, but the absence of mild procoely excludes this specimen from both Diplodocoidea and Lithostrotia. The vertebra cannot be further distinguished from those of a wide range of basal sauropods, cetiosaurids and basal macronarians. However, this plesiomorphic vertebra still signifies the earliest stratigraphic occurrence for a British sauropod dinosaur.

## Introduction

The fossil remains of Middle Jurassic sauropods are extremely rare [1]. The major finds of Middle Jurassic sauropods are in China (*Shunosaurus* [2], *Omeisaurus* [3], *Mamenchisaurus* [4]), Argentina (*Patagosaurus* [5]), North Africa (*Spinophorosaurus* [6], and *Jobaria* [7]).) and Britain (*Cetiosaurus* [8]).

The Middle Jurassic in northeast England has yielded only a few dinosaur bones from the rocks of undoubted Middle Jurassic age [9]. Distal podial elements and vertebral centra were recovered from the Scarborough Formation at White Nab, Scarborough Bay [8,10–11]. The Scarborough Formation is a marine unit within the mainly terrestrial deposits of the Ravenscar Group. The material was identified as both *Cetiosaurus epioolithicus* [12] and *Cetiosaurus longus* [13] in the same year. Neither publication included illustrations and only included the measurements of the centrum: eight inches craniocaudally long and nine inches wide [13]. Upchurch and Martin [8] later re-examined the Scarborough material and determined that it was undiagnosable. Additionally, a single theropod tooth was collected from the marine Coralline Oolite and was tentatively identified as *Megalosaurus* by Williamson [11]. Lastly a femur from a purported megalosaurid dinosaur was collected from the Yorkshire Coast in the 19^th^ Century, but the specimen has since been lost [9].

**Fig 1:**
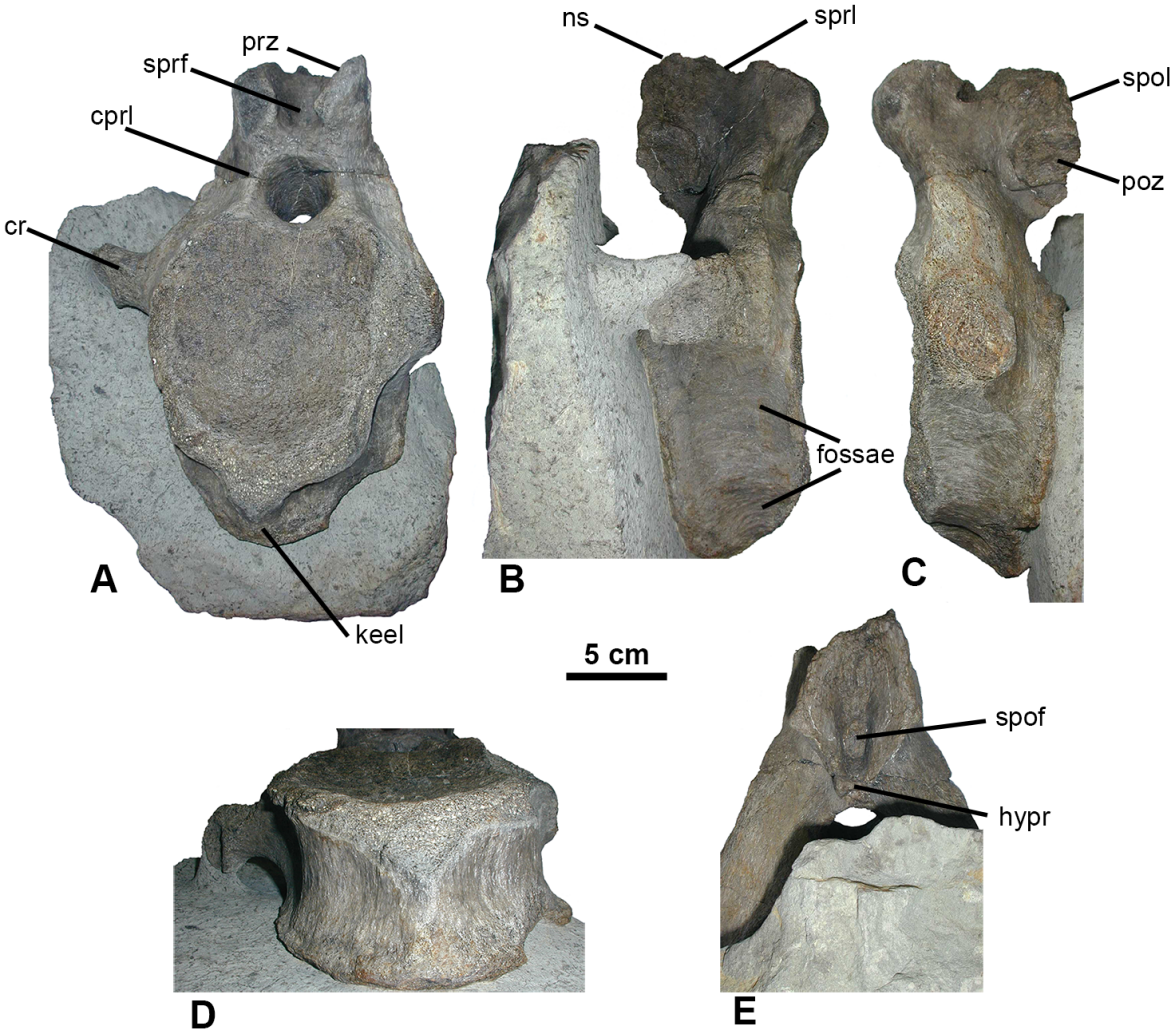
Caudal vertebra, YORYM:2001.9337. Proximal caudal vertebra in oblique view. (A), right lateral (B), left lateral (C), ventral (D) and posterior view of vertebral table (E), Scale = 5 cm. Abbreviations: cr, caudal rib; cprl, centroprezygapophyseal lamina; hypr, hyposphenal ridge; ns, neural spine; poz, postzygapophysis; prz, prezygapophysis; spol, spinopostzygaphyseal laminae; spof, spinopostzygapophyseal fossa; sprl, spinoprezygapophyseal laminae; sprf, spinoprezygapophyseal fossa.

The vertebra described herein (Fig 1) has been collected from the Saltwick Formation located in the basal unit of the Ravenscar Group. The coastal section is formed by rocks of the Middle Jurassic Dogger (~2m thick), Saltwick (~50m thick) and Eller Beck (~4.5m thick) formations. The Dogger Formation rests unconformably on the marine deposits of the Lower Jurassic Whitby Mudstone Formation.

The vertebra is encased in a moderately well-sorted, medium-grained, feldspathic and micaceous quartz sandstone (subarkose). Authigenic clay minerals are abundant, while small nodular siderite and rootlets are also present. This lithotype is relatively common in the non-marine Saltwick Formation, and a broadly similar rock type is present between 5–6 m above the base of the Saltwick Formation at the collection site. The dinosaur vertebra shows little evidence of abrasion and transport. This strongly contrasts with other skeletal material, which is highly abraded, recovered from the younger deposits in the Cleveland Basin [8,11].

Sauropod tracks are known from the Saltwick Formation, but these have not been associated with any vertebrate body fossils to date [14–15]. The extreme rarity of bone material in rocks of the non-marine units of the Ravenscar Group makes the discovery of a body fossil worthy of description, given the paucity of remains in this succession. Undoubtedly, there were diverse dinosaur communities throughout the Middle Jurassic of the Cleveland Basin, based on the rich trace fossil assemblages that have been discovered; yet skeletal elements are very rare [10].

### Geology

The Saltwick Formation is the lowest unit in the Ravenscar Group of the Yorkshire coast (Cleveland Basin). It represents a coastal plain environment complete with river channel, flood plain, lake and marsh deposits (see references in [16]). East of Whitby, the Saltwick Formation is approximately 50 m thick and contains palaeosol and floodplain deposits with crevasse splays and fluvial channels. Saltwick Bay, southeast of Whitby, is the type section for the Saltwick Formation [17]. Non-marine bivalves (*Unio kendalli*), plant fragments and rootlets, and dinosaur tracks are common [14–16,18–22]. The Saltwick Formation overlies the marine Dogger Formation and is succeeded by the marine Eller Beck Formation. The marine dominated Eller Beck consists of two units, a grey mudstone with subordinate sideritic ironstone, with a bed of ooidal ironstone at the base. The upper unit, typically 4–6m thick, consists predominantly of fine to medium-grained sandstone, commonly ripple laminated [17]. Although ammonites are sparse in the Dogger Formation and absent in Saltwick, palynomorph evidence indicates that the latter formation is Aalenian in age [23–25].

## Materials and Methods

The specimen (YORYM:2001.9337, Yorkshire Museum, York UK) is permanently reposited in the collections of The Yorkshire Museum (York Museums Trust, York, UK) and is accessible to all researchers. No permits were required for the described study, which complied with all relevant regulations. The collection of vertebrate and invertebrate fossil remains from below the mean high-tide mark are only restricted in a handful of localities in the UK (e.g. Lyme Regis, Jurassic Coast World Heritage site where local by-laws protect vertebrate fossils), that does not include the site in the current study. The current interpretation of UK law is that fossils that fall out of a cliff (i.e. *ex situ*) may be regarded as having been abandoned by the landowner. Locality and horizon data are described below.

The specimen was studied using X-ray Computed Tomography (CT) in the Department of Radiology at the Manchester Royal Infirmary (University of Manchester, UK). However, the high density of the sample only permitted a bulk density scan yielding limited information on the external geometry of YORYM:2001.9337.

### Systematic Paleontology

Dinosauria Owen 1842 [26]

Saurischia Seeley 1888 [27]

Sauropoda Marsh 1878 [28]

Eusauropoda Upchurch 1995 [29]

Specimen number YORYM:2001.9337, housed in the Yorkshire Museum (York Museums Trust, York, UK). The specimen consists a proximal caudal vertebra (Fig 1).

### Locality and horizon

YORYM:2001.9337 was recovered from a coastal cliff section near the town of Whitby (North Yorkshire, UK). GPS coordinates may be provided on request. The vertebra was recovered from talus deposits at the base of the cliff at Long Bight approximately 600 m east of Whitby East Pier. The sediment that adheres to YORYM:2001.9337 is a fine-grained, well-cemented silty sandstone that bears no resemblance to the lithology of the underlying marine deposited Dogger Formation (typically grey, weathering yellow-brown, ferruginous sideritic sandstone, medium- to coarse-grained, with sporadic phosphatic pebbles; occasional berthierine (chamosite) ooids [17]). Although the source horizon was not definitively located, based on the relative position of the vertebra in the talus slope, resting on top of the Dogger Formation, the vertebra came from the Middle Jurassic, Aalenian (175.6 ± 2.0–171.6 ± 3.0 Ma) Saltwick Formation. The presence of rootlets within the matrix that adheres to the vertebra also supports an origin from within the terrestrial deposits of the Saltwick Formation, given rootlets are not seen in either of the marine Dogger or Eller Beck formations.

### Description and comparison

The vertebra (YORYM:2001.9337) is distinctly amphicoelous with a heart-shaped centrum (Fig 1A). The length of the centrum along the ventral surface is 70 mm and the height of the anterior face of the centrum measures 145 mm. The vertebra is anteroposteriorly short with a length:height ratio of 0.48 (Fig 1B and 1C). The anterior portion is exposed, while the posterior articular facet of the centrum is still mostly encased in matrix (Fig 1B). The anterior and posterior surfaces of the centrum are verified by X-ray computed tomography. The posterior margin of the centrum is marked by a distinct, low, 5 mm wide ‘rim’ (Fig 1B–1D). The ventral surface has a low keel running anteroposteriorly along the centrum; although the anterior portion of this structure has been eroded (Fig 1D). Fossae are present on both the lateral (immediately below the caudal ribs) and lateroventral surface of the centrum (Fig 1B and 1C), defining paired ventro-lateral and ventro-medial ridges anteroposteriorly aligned. There are no pleurocoels on the ventral or lateral surfaces of the centrum. The chevron facets are present but are not prominent (Fig 1D).

One caudal rib is complete and has an expanded, concave, ventrally-facing surface on the distal end of the process (Fig 1A, 1B and 1D). The estimated distance between the tips of the processes is 240 mm. The caudal ribs are positioned dorsally high on the centrum, 120 mm from the ventral edge, and anteriorly forward relative to the mid-plane.

The postzygapophyses are intact (Fig 1C), but the prezygapophyses are slightly damaged (Fig 1A). The pre- and postzygapophyses are steeply angled at 50° and 60° respectively (Fig 1B and 1C). The prezygapophyses are higher than the postzygapophyses, relative to the centrum. The prezygapophyses project beyond the anterior face of the centrum, which in lateral view follows a shallow ‘arc’ starting level with the caudal ribs. Short spinoprezygapophyseal laminae run along the anteriolateral portion of the neural spine and form a small supraprezygapophyseal fossa (Fig 1A). The postzygapophyses appear as facets on the neural spine. The dorsal margins of the postzygapophyses give rise to short spinopostzygaphyseal laminae that meet to form the posterolateral portion of the neural spine (Fig 1B and 1C). The laminae form the lateral margins of a narrow, ‘slot-like’ spinopostzygapophyseal fossa at the base of the neural spine (Fig 1E). A short, hyposphenal ridge is present from the base of the postzygapophyses and runs ventrally to the neural canal. The centroprezygapophyseal lamina ascending from the transverse processes expands and supports the prezygapophyses to form the lateral margin of the neural canal (Fig 1A and 1B).

The neural spine is laterally compressed and anteroposteriorly long. The neural arch is broadly attached to the centrum and centered over the anterior half of the centrum. The neural canal is circular in cross-section, dips posteriorly 30° from horizontal, and expands dorsoventrally from 35 mm to 42 mm in diameter from anterior to posterior opening (Fig 1A). A small fossa is present on the centrum in the neural canal.

## Discussion

The gross morphology of YORYM:2001.9337 is very distinct and clearly conforms to the caudal character of Sauropoda (presence of a hyposphenal ridge) and Eusauropoda (length to width ratio 0.5–0.6) [30]. The length to height ratio of the vertebra, shape of the neural spine, presence of laminae, and the placement of the postzygapophyses on the neural spine are also unique to sauropods [1,30–32]. Furthermore, amphiocoelous vertebrae, anterioposteriorly short centra, caudal ribs and a forward extension of the prezygopophyses beyond the centra, are all typical for anterior caudal vertebrae in basal sauropods (i.e., *Cetiosaurus*, brachiosaurids and basal macronarians) [1]. The plesiomorphic characters, amphiocoelous centra with no pleurocoels, exclude YORYM:2001.9337 from being in more derived sauropod clades [1]. The pronounced ventral keel is unique to this sauropod and contrasts with the midline groove that occurs on the anterior caudals of basal sauropods such as *Vulcanodon*, *Cetiosaurus*, *Omeisaurus*, and *Haplocanthosaurus*, as well as from derived sauropod clades [1]. Thus, the ventral keel represents an autapomorphy that distinguishes this sauropod from other currently known sauropods. However, due to the limited number of described caudal vertebrae synapomorphies within basal sauropods, it is difficult to assess to which sauropod clade YORYM:2001.9337 belonged.

The distinct foramen present in the mid-portion of the floor of the neural canal is a feature recognised in other sauropod proximal caudal vertebra (PLM and VME personal observation), but has largely been unreported. This feature is almost certainly the basivertebral foramina. In extant vertebrates, they are paired foramen separated by a very thin bony septum and house the basivertebral veins. The foramen occurs in the cervical, dorsal, sacral and anterior-most caudal vertebrae of reptiles.

The vertebra (YORYM:2001.9337) is more similar to *Cetiosaurus* than to any other sauropod dinosaur. However, YORYM:2001.9337 is at east 4 million years older than any known *Cetiosaurus* species [8] and has a distinctive keel on the centrum. The centrum of YORYM:2001.9337 displays a shallow concavity on both of the lateral (immediately below the transverse processes) surfaces of the centrum. The concavity is shallower than those in *C*. *brevis* and is not a distinct pleurocoel such as seen in *Diplodocus* and *Barosaurus* [8,33,34]. The neural arch is broadly attached to the centrum and is located more posteriorly than in *C*. *oxoniensis* anterior caudals. YORYM:2001.9337 is very similar to *C*. *brevis* (NHMUK R2544-2547, Natural History Museum, London, UK.) and the Rutland *Cetiosaurus* (LCM G468.1968, Leicester City Museum, Leicester, UK) based on the overall morphology of the centrum; however, *C*. *brevis* and the Rutland *Cetiosaurus* caudal vertebrae lack the ventral keel, distinct anteroposterior ventrolateral ridges and have greater length:height ratios [8,35]. Furthermore, the caudal ribs of the Whitby vertebra are more anteriorly positioned on the centrum than in *C*. *brevis* [8].

Similarities between the Whitby sauropod and *Haplocanthosaurus delfsi*, CM572 from the Morrison Formation [36] are also prominent. The centra of *H*. *delfsi* are amphiocoelous and lack pleurocoels. The length to height ratio is very similar to YORYM:2001.9337, especially among caudal vertebrae 2 (0.44), 3 (0.45) and 4 (0.42). However, the first two caudal vertebrae in *H*. *delfsi* have wing-like processes on the caudal ribs, which is a common feature among diplodocids [36].

The proximal caudal vertebra of *Patagosaurus fariasi* from Argentina (Callovian), is also comparable to YORYM:2001.9337. They both possess laterally compressed neural spines, short prezygopophyses and the postzygopophyses separated by spinopostzygapophyseal fossa, however, *P*. *fariasi* lacks the keel that is distinct to YORYM:2001.9337 and the postzygopophyses are more prominent in the former [5,37].

## Conclusions

The vertebra YORYM:2001.9337 is described here as representing a eusauropod dinosaur based on the short high centrum and the presence of the spinopostzygopophyseal laminae[29] (Fig 1). Whilst the morphology of the vertebra is distinct and possesses some diagnostic characters, most notable being the anteroposteriorly aligned keel that is prominent on the ventral surface of the centrum, a single centrum is not sufficient to justify description as a new sauropod taxon.

The taxonomy and phylogenetic relationships among British cetiosaurid sauropods has not always been clear [8]. However, valid British cetiosaurs range between the Bajocian to Bathonian [8]. Thus, YORYM:2001.9337 is significantly older than recognised *Cetiosaurus* remains by at least four million years. The presence of YORYM:2001.9337 in the Middle Jurassic (Aalenian) increases our knowledge on the occurrence of eusauropods in a period of time that provides scant body-fossil evidence for this group of dinosaurs.
